# Variability in innate host immune responses to cryptococcosis

**DOI:** 10.1590/0074-02760180060

**Published:** 2018-04-12

**Authors:** Mariam Garelnabi, Robin C May

**Affiliations:** University of Birmingham, Institute of Microbiology and Infection, School of Biosciences, Edgbaston, Birmingham, UK

**Keywords:** Cryptococcus neoformans, host-pathogen interactions, innate immunity, infection, pathogenesis, macrophages, host variation

## Abstract

Cryptococcosis is an invasive fungal disease caused by *Cryptococcus neoformans* and the closely related species *C. gattii.* The severe form of the disease, cryptococcal meningitis (CM), is rapidly fatal without treatment. Although typically a disease of immunocompromised (especially HIV-positive) individuals, there is growing awareness of cryptococcal disease amongst non-immunocompromised patients. Whilst substantial progress has been made in understanding the pathogenicity of *C. neoformans* in HIV patients, prospective data on cryptococcosis outside the context of HIV remains lacking. Below we review how innate immune responses vary between hosts depending on immunological status, and discuss risk factors and predictors of disease outcome in different groups.

Cryptococcal meningitis (CM) remains the leading cause of fungal meningitis worldwide, afflicting up to 1 million individuals annually with approximately 600,000 subsequent deaths (Park et al. 2009). This disease arises following severe infection with members of the genus Cryptococcus in a variety of human hosts with different immune backgrounds. The majority (80%) of cryptococcosis cases are HIV-associated, and are caused by *Cryptococcus neoformans*; placing this disease as the second most common cause of AIDs-related deaths after tuberculosis (Park et al. 2009, Jarvis et al. 2010, Rajasingham et al. 2017). *C. neoformans* also causes CM in non-HIV, immunocompromised patients and at a rising frequency in ‘otherwise healthy’ individuals (Zhu et al. 2010, George et al. 2018).

Infection begins upon inhalation of cryptococcal spores from the environment, triggering the innate immune system. Macrophages play an integral role in anti-cryptococcal defense, with alveolar macrophages acting as first responders in the lungs where they detect and engulf cryptococcal spores (Giles et al. 2009). As intracellular pathogens, cryptococci are capable of survival and replication within host macrophages (Levitz et al. 1999, Sabiiti et al. 2014); failure to clear pulmonary infection leads to fungal dissemination throughout the body and towards the brain resulting in CM via the Trojan horse model.

Whilst early studies of the manifestations of cryptococcosis have shown clinical and prognostic differences between the infecting species (Speed and Dunt 1995), more recent investigations into the pathogenesis of *C. neoformans* in particular have revealed a critical role for host immune status in conferring protection from, or controlling disease progression towards, meningitis (Chen et al. 2000). This has led to the division of *C. neoformans* patients into three groups: HIV-associated; Non-HIV immunocompromised patients; and ‘otherwise healthy’ immunocompetent individuals.

Hosts with intact immune systems mount an immune response that leads to clearance of the infection, or the establishment of a latent, asymptomatic infection accompanied by the formation of cryptococcomas. Patients with impaired cell-mediated immunity are unable to effectively clear *C. neoformans.* Thus, effective innate immune activation and a sufficient inflammatory response are key to the control of cryptococcosis. Below, we review how underlying host innate immune responses vary between the aforementioned three groups of human hosts in response to cryptococcal disease caused by *C. neoformans*. We also assess current understanding of how immune responses in different hosts may be predictive of protection from, or susceptibility to, CM. For a discussion of the role of adaptive immunity in *C. neoformans* infection, we would refer readers to the recent review by Mukaremera and Nielsen (2017).

*Immune responses in non-immunocompromised individuals* - During the cryptococcosis outbreak in Vancouver 2003 that affected primarily non-immunocompromised individuals, *C. gattii* was identified as a primary pathogen of the healthy. However, increased recognition of cryptococcosis cases due to *C. neoformans* in other immunocompetent patients provides strong evidence that this species also harbors capabilities as a primary pathogen, despite early classification as a strictly opportunistic pathogen.

In ‘otherwise healthy’ individuals, pulmonary infection is generally asymptomatic. The pattern recognition receptor (PRR) CD14 in association with Toll-like receptor 4 (TLR4), TLR2 (Shoham et al. 2001), and CD18 (Dong and Murphy 1997) on the surface of macrophages recognise the cryptococcal capsular polysaccharide glucuronoxylomannan (GXM), driving localised immune recognition and enhanced phagocytosis (Dong and Murphy 1997, Yauch et al. 2004, Giles et al. 2009, Levitz 2010, Garcia-Rodas and Zaragoza 2012). The macrophage arsenal to eliminate engulfed fungi includes the release of proinflammatory cytokines and chemokines that extend cell mediated immunity by increasing monocyte and neutrophil recruitment to the site of infection, and antigen presentation to T-cells. Secretion of these cytokines typically follows induction of the nuclear factor-κΒ (NF-κΒ) pathway (Figure) that is regulated by microRNAs (miRNAs) (Chen et al. 2017) to promote the expression of inflammatory cytokines in attempts to kill the pathogen (Johnston and May 2013, Smith et al. 2015). However, this pathway can be modulated by both shed GXM and live cryptococci via different mechanisms depending on the stage of infection (Hayes et al. 2016). Early on in the infection, GXM acts as an anti-phagocytic cloak for *C. neoformans* (Feldmesser et al. 2000). Following ingestion by macrophages, cryptococci continue to produce and shed the polysaccharide (Feldmesser et al. 2000), which has been shown to reduce LPS-associated proinflammatory cytokine responses both *in vitro* (Monari et al. 2006) and in a murine infection model by inhibiting MyD88 activation following its interaction with the FcγRIIB receptor (Figure) (Monari et al. 2006, Piccioni et al. 2013). Furthermore, Hayes et al. (2016) recently showed in RAW 264.7 murine macrophages that extracellular GXM inhibited LPS-induced nuclear translocation of the p65 protein, while intraphagosomal GXM increased the duration of nuclear translocation of the p65 and ΙκΒα proteins, consequentially eliminating the expression of TNFα or inducible NOS (iNOS).

**Figure:**
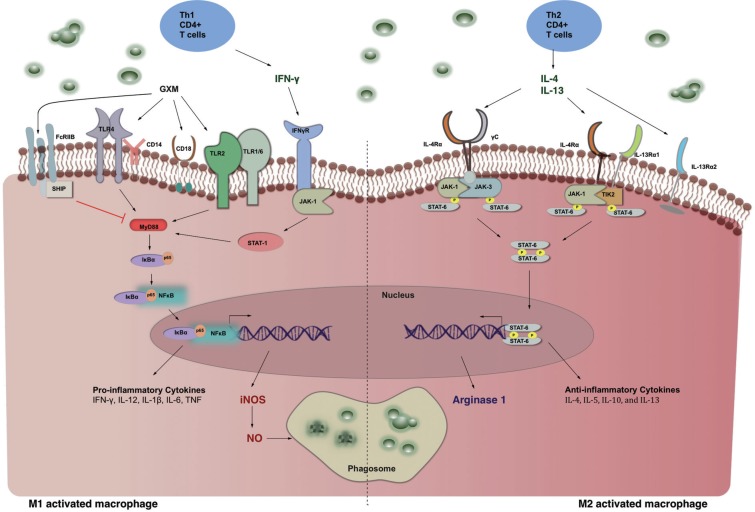
Macrophage activation state determines the outcome of *Cryptococcus neoformans* clearance.

The primary defense against cryptococcosis in immunocompetent hosts is orchestrated by Th1-type CD4^+^ T-cells (Kawakami et al. 1995, Wozniak et al. 2009, Olszewski et al. 2010) that produce interferon-γ (IFN-γ), and direct macrophages towards a Th1, classically activated, phenotype (Kawakami et al. 1995). Th1 responses have been associated with increased fungal clearance, whilst Th2 responses are associated with poor anticryptococcal defenses (Figure); thus, the interplay between Th1 and Th2 host responses during cryptococcal infection is a major determinant of disease outcome (Koguchi and Kawakami 2002, Arora et al. 2011). Host susceptibility studies in mice have shown that production of the Th1-type cytokines IFN-γ and IL-12, as well as the pro-inflammatory cytokine TNF-α confers protection against cryptococcosis (Kawakami et al. 1996a, Kawakami et al. 1996b, Kawakami et al. 1996c, Decken et al. 1998, Herring et al. 2002). Conversely, production of Th2-type cytokines (including IL-4, IL-5, IL-10, and IL-13) renders hosts vulnerable to severe disease and mortality (Muller et al. 2007). GXM has also been shown to directly inhibit T-cell proliferation in mice, leading to dampened Th1 responses, and diminished resolution of the infection (Yauch et al. 2006).

Macrophage differentiation is regulated by granulocyte-macrophage colony-stimulating factor (GM-CSF). The presence of anti-GM-CSF autoantibodies in the spinal CSF inhibits the activity of GM-CSF, and has been associated with poor disease prognosis in cases of CM due to *C. neoformans* in otherwise healthy hosts (Rosen et al. 2013). Further attempts to characterise predispositions to CM have identified genetic background as a risk factor for developing severe cryptococcosis. Polymorphisms in the Fc gamma receptor (FcγR) (Meletiadis et al. 2007, Hu et al. 2012) and mannose-binding lectin (MBL) (Ou et al. 2011) have been shown to increase susceptibility to cryptococcal infections in immunocompetent individuals.

A retrospective study by Nadrous et al. (2003) of cryptococcosis in 42 immunocompetent hosts showed that 86% of patients had isolated pulmonary cryptococcosis with no evidence of fungal dissemination to the CNS, of which 33% were asymptomatic, and 92% were able to resolve the infection either with or without antifungal treatment or surgical abscission. Whilst no quantitation of innate immune responses to infection at the individual level were performed, these findings show that the majority of cryptococcosis patients with previously intact immune systems and no evidence of disseminated disease are able to effectively eliminate cryptococcal infection.

*Immune responses in HIV patients* - Cryptococcal meningitis is the second largest cause of mortality among HIV patients worldwide. Disease prevalence became more apparent among immunocompromised patients particularly during the HIV pandemic in the 1970s; where 80% of CM sufferers were HIV-seropositive. However, as of 2014, the frequency of cryptococcal disease among this group has dropped to a third (Mirza et al. 2003, Cox et al. 2014). Thanks to the widespread of anti-retroviral (ARV) therapies, it was estimated that 223,100 cases of CM in HIV patients occur each year worldwide, causing 181,100 deaths annually (Rajasingham et al. 2017).

T-cell depletion in HIV patients is a determining immunological feature that renders this group susceptible to CM. However, in a recent study, Neal et al. (2017) demonstrated in a murine model that whilst CD4^+^ T-cells assist in the clearance of the fungal disease, they also contribute to disease dissemination in the CNS as well as tissue damage as a result of immunotherapy, leading to immune reconstitution inflammatory syndrome (IRIS). Reduced counts of CD4^+^ T-cells in this patient group conventionally results in the proliferation of CD8^+^ T-cells and increased recruitment of myeloid cell lineages to combat ongoing infection, subsequently affecting clinical outcomes of disease and deaths from CM.

Other reports have identified a number of protective immune traits and markers of susceptibility to acquiring CM in HIV-seropositive patients. Cerebral spinal fluid (CSF) cytokine profiles can reliably report disease progression and predict mortality in HIV patients. Data from several patient cohorts agree that inflammatory and Th1 cytokine responses confer protective advantages in HIV patients (Jarvis et al. 2014, Jarvis et al. 2015, Scriven et al. 2017). HIV-seropositive hosts with high CSF levels of interleukin (IL)-6, interferon-γ (IFN-γ), IL-8, IL-12, TNF-α and CXCL10 show improved fungal clearance and survival rates; while increased levels of IL-4 and IL-10 have been associated with high serum levels of cryptococcal GXM that impairs monocyte activation and cripples cell-mediated responses to infection - effectively predicting mortality in this group of patients (Retini et al. 1998, Jarvis et al. 2015, Mora et al. 2015). However, low T-cell infiltration of the CSF rather than alternative macrophage activation is a major determinant of disease outcome in HIV-associated cryptococcosis (Scriven et al. 2017). Interestingly, not all HIV patients with low CD4^+^ T cell counts develop cryptococcosis. This is thought to be largely due to the contribution of genetic factors to disease susceptibility. Following the association of polymorphisms in the FcγR with susceptibility to cryptococcosis in non-HIV patients (Meletiadis et al. 2007, Ou et al. 2011, Hu et al. 2012), Rohatgi et al. (2013) showed that HIV patients who were either heterozygous or homozygous for the FCGR3A 158V allele, were 2.1- and 21-fold at higher risk of developing cryptococcal disease than individuals without the allele, respectively. The group suggested that increased recognition of *C. neoformans* and immune activation leading to a rise in phagocytic uptake as the functional implication of this allele. These findings provide an alternative approach for identification of at-risk individuals, and more personalised treatment strategies.

*Immune responses in non-HIV, immunocompromised patients* - This group of patients is highly heterogeneous, encompassing a range of patients from solid organ transplant (SOT) recipients to those with underlying immune defects such as sarcoidosis, diabetes mellitus, and patients receiving anti-cancer treatments (Singh et al. 2009). Similar to HIV-sufferers, organ transplant recipients also present with defects in T-cell-mediated immune responses to cryptococcosis due to immunosuppression (Pappas et al. 2001).

In SOT patients, the severity of infection has been associated with the level of immunosuppression (Husain et al. 2001). Studies by Pappas et al. (2001) have shown that the type of immunosuppressive treatment(s) administered to this group influences their susceptibility to CNS-associated cryptococcosis, responses to antifungal treatments, and likely fatality. Another study by Saha et al. (2007) provided serological evidence that the majority of cryptococcal infections in these patients were due to reactivation of pre-transplantation infections. Individuals with circulating antibodies against *Cryptococcus* prior to organ transplantation acquired cryptococcosis sooner than those without previous exposure to the pathogen, and were more likely to develop CM. Disease progression in this subset was also dependent on the type of organ transplanted, although transmission of infection from organ donor to recipient has rarely been observed (Singh et al. 2009).

*Discussion* - Whilst the reduction in global HIV-associated CM is testament to improved ART and antifungal treatment strategies, the rise in frequency of non-HIV and non-transplant associated cryptococcosis in the developed world represents a cause for concern. HIV-associated CM remains the most extensively investigated among the three groups. Whilst the majority of cryptococcal infections in previously healthy individuals are asymptomatic, clinical manifestations and prognoses vary greatly. Current clinical guidelines for this group are generated from immunocompromised cohorts and thus may not be appropriate for disease management in immunocompetent hosts. Whilst numerous case reports of ‘unusual’ cryptococcosis cases have been published, there is a scarcity of prospective data on the management of CM in previously healthy individuals.

A recent article by Pirofski and Casadevall (2017) discussed the effects of host-mediated damage on the progression of cryptococcosis. Given the variety of host-microbe interactions that occur within the different patient groups discussed above, and the associated alteration of the inflammatory immune responses, this damage-response framework (DRF) model may be particularly helpful for understanding infections in otherwise healthy individuals. Clearly, however, there is currently a lack of characterisation studies in non-HIV cryptococcal patients with which to inform this model.

It has previously been shown that cytokine expression by macrophages is not permanently biased to either Th1 or Th2 responses, and may be modulated (Arora et al. 2011); hence, the fluidity in macrophage activation states instigates varied responses in individuals with robust immune systems (Koguchi and Kawakami 2002). Furthermore, the genetic contribution to phenotypic challenges in anti-cryptococcal responses among cryptococcosis sufferers provides an additional tool for early diagnosis in at-risk cohorts. However, difficulties arise in the case of non-HIV immunocompetent hosts who may not be considered at-risk and therefore subject to presumptive genetic testing in comparison to their immunocompromised counterparts. In addition, it was brought to light in a recent article by Netea (2013), that the association of the above-mentioned polymorphisms with susceptibility to severe cryptococcosis is not descriptive of all patients across different cohorts depending on ethnic backgrounds.

Whilst therapeutic approaches for HIV-associated cryptococcal disease in combination with ARTs are successfully being established, efforts to determine protective features of the innate immunity in non-HIV-associated disease are still underway. Knowledge of cryptococcal pathogenesis remains minimal in patients with known immunological defects beside HIV, and treatment strategies remain unspecific. This may be at least partially due to the inappropriate clustering of these two groups together into a single “non-HIV-associated cryptococcosis” category, when in fact they represent at least two distinct cohorts. A better understanding of the sources of variation between and within patient groups is urgently needed in order to help inform strategies to appropriately modulate the immune responses at the level of the individual to improve disease outcomes.
